# Injuries and illness of athletes at the Tokyo 2020 Olympic and Paralympic summer games visiting outside facilities

**DOI:** 10.1016/j.smhs.2024.01.003

**Published:** 2024-01-17

**Authors:** Shuji Sakanashi, Hideharu Tanaka, Hiroyuki Yokota, Yasuhiro Otomo, Tomohiko Masuno, Kousuke Nakano, Junichi Inoue, Manabu Sugita, Takahiko Tokunaga, Nagisa Kato, Tomoya Kinoshi, Hironori Inoue, Hiroto Numata, Koshi Nakagawa, Ryo Sagisaka, Shota Tanaka, Tetsuya Miyamoto, Takao Akama

**Affiliations:** a The Tokyo Organising Committee of the Olympic and Paralympic Games, Tokyo, Japan; b Department of Sports Medicine Kokushikan University, Tokyo, Japan; c Graduate School of Emergency Medical System, Kokushikan University, Tokyo, Japan; d Research Institute of Disaster Management and EMS Kokushikan University, Tokyo, Japan; e Graduate School of Health and Medical Science, Nippon Sports Science University, Japan; f Tokyo Medical and Dental University Hospital, Trauma and Acute Critical Care Center, Tokyo, Japan; g Graduate School of Medicine, Nippon Medical University, Tokyo, Japan; h Saitama City Hospital, Emergency Department, Saitama, Japan; i Graduate School of Medicine, Juntendo University, Tokyo, Japan; j Emergency Lifesaving Academy Tokyo, Tokyo, Japan; k Nippon Medical School Musashikosugi Hospital, Emergency and Critical Care Center, Knagawa, Japan; l Department of Integrated Science and Engineering for Sustaiable Society, Chuo University, Japan; m Faculty of Sport Sciences, Waseda University, Saitama, Japan; n National Hospital Organization Disaster Medical Center, Tokyo, Japan

**Keywords:** Olympic games, Paralympic games, Sports injuries, Illness, Heat-related illness, 2020 Tokyo

## Abstract

This study aimed to identify the reasons for transferring athletes to local medical facilities during the Olympic and Paralympic Games. Data on 567 injuries and other illnesses of athletes treated at the on-site clinics were collected from the Tokyo 2020 Organizing Committee. Of these, 84 athletes who required outpatient care during the Games were registered for this survey. During the Olympic and Paralympic Games, 66 (8.3/1 000) and 18 (7.2/1 000) athletes, respectively, consulted external medical facilities. In the Olympic Games, the reasons for these visits included 48 cases (72.7%) of injuries, 13 (19.7%) cases of illnesses, and 5 (7.6%) cases of heat stroke illness (HSI). Of these patients, 56 (84.9%) were treated as outpatients and 10 (15.1%) were hospitalized, while three of these patients required hospitalization for > 7 days. On the other hand, in the Paralympics Games, there were 7 (38.8%) cases of injuries, 9 (50.0%) other illnesses, 1 (5.6%) case of HSI, and 1 (5.6%) other cases, of which 11 (61.1%) were treated as outpatients and 7 (38.9%) were hospitalized, but none was hospitalized for > 7 days. Injuries accounted for 70% of the total cases at the 2021 Olympic Games, but only three (0.05%) were severe cases that required hospitalization for more than 1 week. In contrast, in the Paralympic Games, other illnesses accounted for approximately half of the total cases. This study provides details on the extent of injuries and other illnesses that were transferred to outside facilities, which has not been documented in previous games.

## Abbreviations

Tokyo 2020 Games: The Tokyo 2020 Olympic and Paralympic Games
HIS: heat stroke illness
EMR: electronic medical registration
IOC: International Olympic Committee
BMX: bicycle motocross
COVID-19: Coronavirus disease
*SD*: standard deviation
ER: emergency room
*n*: numbers

## 1 Introduction

The Tokyo 2020 Olympic and Paralympic Games (Tokyo 2020 Games) were attended by 11 ​420 Olympic athletes from 206 countries and 4 403 Paralympic athletes from 163 countries, respectively.^1^ Medical infrastructures for the games included two clinics for athletes and spectators, and several ambulances at all 43 competition venues. Efforts were made to ensure that appropriate medical care was provided to athletes and staff^1^; however, if medical treatment had to be continued beyond the clinics at each venue or at the athletes' village, the athletes were transported to designated game hospitals for treatment at external facilities. Athletic injuries and illnesses at the Olympics have been reported in detail since the 1990s.^2^, ^3^, ^4^, ^5^, ^6^ Injuries rates at the recent Summer Olympic Games were reported in Rio de Janeiro 2016 (8%),^2^ Beijing 2008 (10%),^3^ and London 2012 (11%).^5^ Meanwhile, disease outbreaks were reported as 651 (5%) cases at Rio de Janeiro Olympic Games 2016,^2^ as well as at London Olympic Games 2012 (7%).^5^ Furthermore, city of Tokyo, the main venue for the Games, has experienced a heat island effect, and there was a concern about the occurrence of heat stroke illness (HSI).^7^^,^^8^ The occurrence of HSI, especially in endurance sports such as marathon running and competitive walking, has become a major problem in hosting the Summer Olympic Games. However, there are no detailed reports on visits to outside facilities, excluding the medical unit at the games; hence, this aspect is not clear. The purpose of this study was to clarify the status of athletes' visits outside facilities during the Tokyo 2020 Games. We believe that this study will help the medical system of international mass gathering sports events in the future.

## 2 Methods

### 2.1 Study design

This retrospective cohort study used data on 84 external medical facility visits collected from the Tokyo Organizing Committee for the Olympic and Paralympic Games.

### 2.2 Study subjects and data extraction

The records provided by the Medical Services Department of the Organizing Committee were categorized. The following information was obtained: 1) medical operation managers’ daily reporting data (the number of cases reported by the medical operation manager at each venue); 2) Japanese version of Surveillance in Post Extreme Emergencies and Disaster (known as J-SPEED) data (non-athlete reported data); 3) electronic medical registration (EMR) data (athlete treatment records); 4) data on external medical facility visits; 5) main operation center data (information from the medical coordination headquarters); and 6) final diagnosis information from the treating hospital. This study was conducted using data from visits to external medical facilities.

Data on visits to external medical institutions were based on a system in which medical institutions and organizing committees collaborated to report the details of visits, and the data collected from 244 cases were analyzed. Subsequent analyses were conducted on 84 cases that occurred between July 21 and August 8, 2021 (during the Olympic Games) and between August 24 and September 5, 2021 (during the Paralympic Games).

**Fig. 1:**
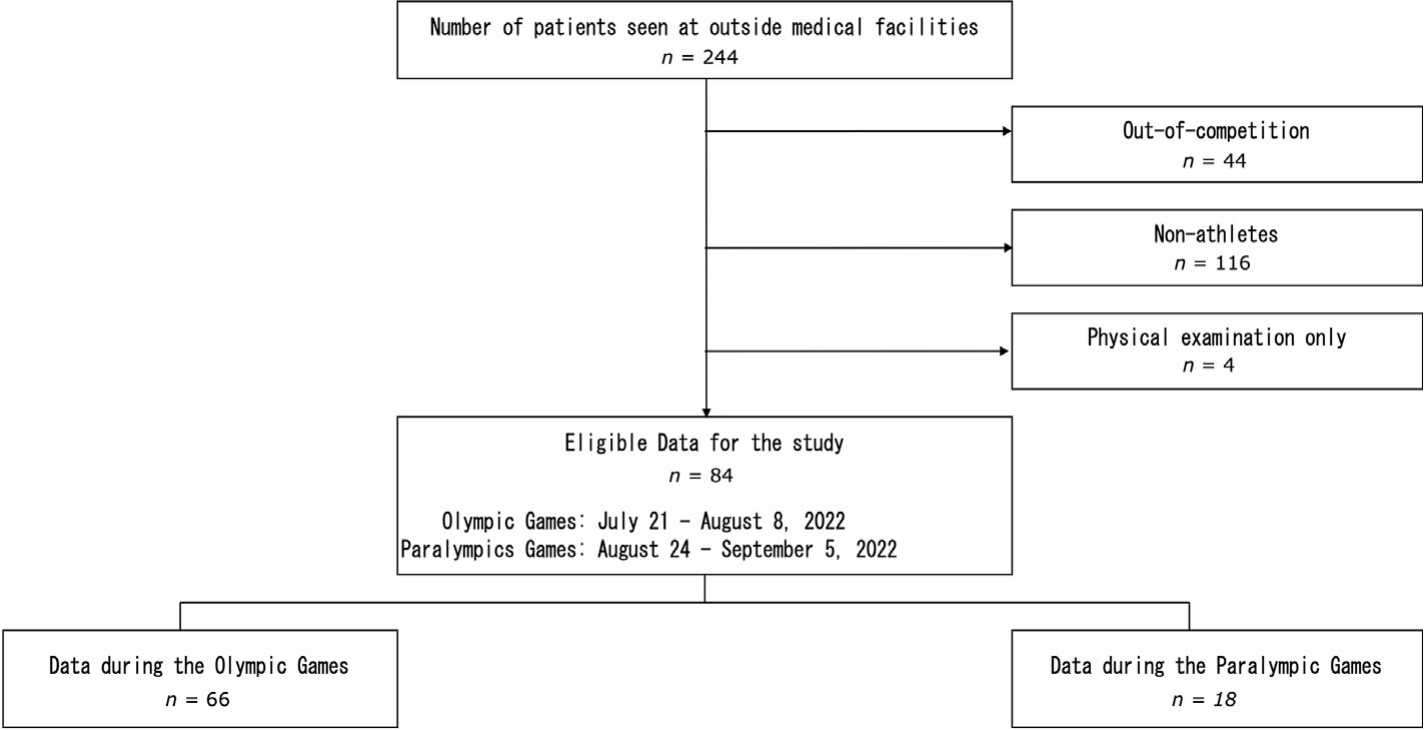
Target data and inclusion criteria.

The exclusion criteria were as follows: (1) out-of-period data (*n* ​= ​40); (2) non-athletic data (*n* ​= ​116); and (3) visits for examination purposes only (*n* ​= ​4). A total of 160 cases were excluded, as were data from these athletes' external medical visits that occurred during the competition (Fig. 1).

### 2.3 Data components

The following data were recorded during visits to external medical institutions provided by the MSD of the Organizing Committee: Data on (1) date of outpatient visit, (2) competition name, (3) gender, (4) age, (5) place of occurrence, (6) circumstances of occurrence, (7) name of injuries or diseases, (8) means of transport, (9) severity, and (10) presence or absence of hospitalization as well as the duration of hospitalization were extracted. The following three categories were used to classify the reasons for the visit: (1) injuries, (2) illness, and (3) heat stroke illness (HSI).

### 2.4 Definition of injuries classification and severity

Based on the medical records at the time of visitation, the reasons for the visits were classified as “ injuries,” “illness,” or “HSI”. In accordance with the International Olympic Committee (IOC) consensus statement,^9^ an injury was defined as tissue damage and derangement of normal physical function due to participation in sports. Illness was defined as physical pain or disability unrelated to injuries, no history of injuries, and medical treatment or chief complaint in the medical record at the time of the visit. The patients’ severity classifications and destination hospitals were determined by an athlete clinic physician at the venue. In terms of severity classification, “mild” was defined as those cases that were only observed and did not require hospitalization, “moderate” as those that required hospitalization and were treated within 1 week, and “severe” as those that required emergency transportation to a tertiary care hospital or required surgery and hospitalization for 1 week or longer.

### 2.5 Statistical analysis

Inpatient background characteristics and continuous variables are represented as mean (standard deviation), and categorical variables are represented as the number of cases (%). The incidence of injuries for each sport was calculated as the rate per 1 000 athletes in the competition to the number of injuries. JMP prover 13.2.1 software (SAS Institute, Cary, NC, USA) was used for statistical analyses.

### 2.6 Ethical considerations

In this study, medical data were obtained from the Organizing Committee and used after de-identification of the subjects’ personal information. Additionally, an application for secondary use of the data was submitted to the Ethics Committee on Research Involving Human Subjects of Kokushikan University, and approval for the implementation of the research was obtained (approval number: 21033).

## 3 Results

### 3.1 Patients’ background characteristics

Table 1 summarizes the background data of the 84 athletes who visited outside facilities during the Tokyo 2020 Games. In the Olympic Games, 66 patients (0.6% of all participating athletes) visited an external medical facility, with an average age of (26.9 ​± ​4.4) years, among which 39 (59.1%) were male and 27 (40.9%) were female. In the Olympic Games, 37 (56.1%) athletes were transported by ambulance and 29 (43.9%) were transported by other means (cabs, conventioneers, etc.). According to the reasons for the visits, 48 (72.7%) cases were due to injuries, 13 (19.7%) were due to other illnesses, and 5 (7.6%) were due to heat stroke, with 10 (13.5%) athletes requiring hospitalization and treatment. Moreover, at the Paralympic Games, 18 athletes (0.4%) (average age, [36.9 ​± ​10.5] years), 11 (61.1%) of whom were male and 7 (38.9%) were female, visited external medical facilities. Among these athletes, 11 (61.6%) were transported by ambulance and 7 (30.8%) by other means (taxi, conventioneers, etc.). Furthermore, according to the reasons for the visit, 7 (38.9%) were due to injuries, 10 (55.6%) were due to other illnesses, and 1 (5.6%) athlete had HSI, requiring hospitalization and treatment (Table 1).

**Table 1:** Patients’ background.

	Number of hospital visits			
*n* (%)	Olympic (*n* ​= ​66)		Paralympic (*n* ​= ​18)	
Age (years), mean (*SD*)	26.9	(4.4)	36.9	(10.5)
Gender				
male, *n* (%)	39	(59.1)	11	(61.1)
female, *n* (%)	27	(40.9)	7	(38.5)
Means of transport				
ambulance, *n* (%)	37	(56.1)	11	(61.1)
Non-ambulance, *n* (%)	29	(43.9)	7	(61.1)
Injuries and Illness classification				
Injuries, *n* (%)	48	(72.7)	7	(38.9)
Illness, *n* (%)	13	(19.7)	10	(55.6)
HSI, *n* (%)	5	(7.6)	1	(5.6)
Severity				
Severe, *n* (%)	7	(10.6)	1	(5.6)
Moderate, *n* (%)	4	(6.1)	6	(33.3)
Mild, *n* (%)	55	(83.3)	11	(61.1)
Outcome				
Hospitalization, *n* (%)	10	(15.2)	7	(38.9)
Outpatient clinic/ER, *n* (%)	56	(84.8)	11	(61.1)

SD, standard deviation.

ER, emergency room.

HSI, heat stroke illness *n*, numbers.

### 3.2 Data on injuries and illness incidence and severity classified according to the various competitions during the Olympic Games

Table 2 shows the incidence of external medical consultations per 1 000 athletes in the Olympic Games, as well as the classification of injuries, severity of illness, and length of hospitalization.

**Table 2:** Visits to outside facilities by olympic sport discipline.

				Injuries and Illness classification, (*n*)			Severity, (*n*)			Length of hospitalization, (*n*)			
Olympic sport	Number of occurrences	Athletes (*n*)	Incidence rate per 1 000	Injuries	illness	HSI	Mild	Moderate	Severe	< 1 day	1 day	≤ 7 days	＞ 7 days
**Soccer**	17	616	27.6	16	1		15	2		15	2		
**Boxing**	6	289	20.8	3	3		5	1		5			1
**Cycling-Track-**	6	195	30.8	5		1	5		1	5			1
**Track-and-field**	3	1 670	1.8		2	1	3			3			
**Judo**	5	531	9.4	4	1		4		1	4		1	
**Cycling-Road-**	4	201	19.9	3	1		3	1		3		1	
**Basketball**	2	288	6.9	2					2		1		1
**Marathon**	3	368	8.2			3	3			3			
**Triathlon**	2	109	18.3	2			2			2			
**Swimming**	1	286	3.5		1		1			1			
**Sailing**	2	300	6.7	2			2			2			
**Rugby**	2	312	6.4	2			2			2			
**Handball**	2	359	5.6	2			2			2			
**Volleyball**	2	288	6.9	1	1		1		1	2			
**Skateboarding**	1	80	12.5	1			1			1			
**Baseball**	1	748	1.3	1			1			1			
**Canoeing**	1	163	6.1	1			1			1			
**Weightlifting**	1	197	5.1		1		1			1			
**Cycling-BMX-**	1	66	15.2	1					1			1	
**Shooting**	1	356	2.8		1		1			1			
**Modern pentathlon**	1	72	13.9	1			1			1			
**Wrestling**	1	289	3.5	1			1			1			
**Tennis**	1	190	5.3		1				1		1		
**Total**	**66**	**7 973**	**8.3**	**48**	**13**	**5**	**55**	**4**	**7**	**56**	**4**	**3**	**3**

HIS, heat stroke illness; *n*, numbers.

The sport with the highest incidence of visits to external medical facilities was track cycling (*n* ​= ​6, 30.8 per 1 000), followed by soccer (*n* ​= ​17, 27.6 per 1 000), and boxing (*n* ​= ​6, 20.8 per 1 000). Regarding the incidence of injuries in the various sports, soccer had the highest incidence (*n* ​= ​16), followed by cycling track events (*n* ​= ​5), and judo (*n* ​= ​4). For illness, three cases occurred in boxing (*n* ​= ​3), followed by athletics (*n* ​= ​2). HSI occurred most frequently in marathons (*n* ​= ​3), followed by bicycle track events (*n* ​= ​1), and judo (*n* ​= ​1). In terms of severity, seven cases (16%) were severe, four (6.1%) were moderate, and 55 (83.3%) were mild, with two of the seven severe cases occurring in basketball, one each in other sports involving tennis, bicycle motocross (BMX), volleyball, judo, and bicycle track events. Among the seven severe cases, five injuries were sustained due to contact during the game (two cervical cord injuries, two intracranial hemorrhages, and one case of shoulder dislocation that required surgery), two cases of impaired consciousness, and two cases of illness due to appendicitis.

### 3.3 Data on injuries and illness incidence and severity classified according to the various competitions during the Paralympics Games

Table 3 shows the incidence of external medical consultations per 1 000 athletes in the Paralympic Games as well as the classification of injuries, severity of illness, and duration of hospital stay.

**Table 3:** Visits to outside facilities by paralympic sport discipline.

				Injuries and Illness classification, (*n*)			Severity, (*n*)			Length of hospitalization, (*n*)			
paralympic sport	Number of occurrences(*n*)	Athletes	Incidence rate per 1 000	Injuries	Illness	HSI	Mild	Moderate	Severe	< 1 day	1 day	≤ 7 days	＞ 7 days
**Swimming**	5	605	8.3		5		3	2		3	1	1	
**Athletics**	3	1 078	2.8	1	1	1	3			3			
**Cycling-Road-**	4	233	17.2	4			1	3		1	2	1	
**Blind football**	1	78	12.8		1		1			1			
**Powerlifting**	2	178	11.2	2			1	1		1		1	
**Wheelchair fencing**	1	96	10.4		1		1			1			
**Wheelchair tennis**	1	104	9.6		1				1			1	
**Cycling-Track-**	1	120	8.3		1		1			1			
**Total**	**18**	**2 492**	**7.2**	**7**	**10**	**1**	**11**	**6**	**1**	**11**	**3**	**4**	**0**

HIS, heat stroke illness; *n*, numbers.

Swimming (*n* ​= ​5, 8.3 per 1 000) was the sport with the highest number of visits outside facilities. The next most common sports were cycling (*n* ​= ​4, 17.2 per 1 000) and track and field (*n* ​= ​3, 2.8 per 1 000). In terms of injuries categories, the most frequently occurring sport among injuries was cycling road racing (*n* ​= ​4), followed by powerlifting (*n* ​= ​2), and track and field (*n* ​= ​1). In terms of illness, most cases occurred in swimming (*n* ​= ​5), followed by one case each in track and field, ball indoor soccer, wheelchair fencing, and wheelchair tennis. In HSI, the only occurrence was in athletics. In terms of severity classification, one case (5.6%) was classified as severe, six cases (33.3%) as moderate, and 11 cases (61.6%) as mild.

### 3.4 Classification of injuries and other illnesses

**Fig. 2:**
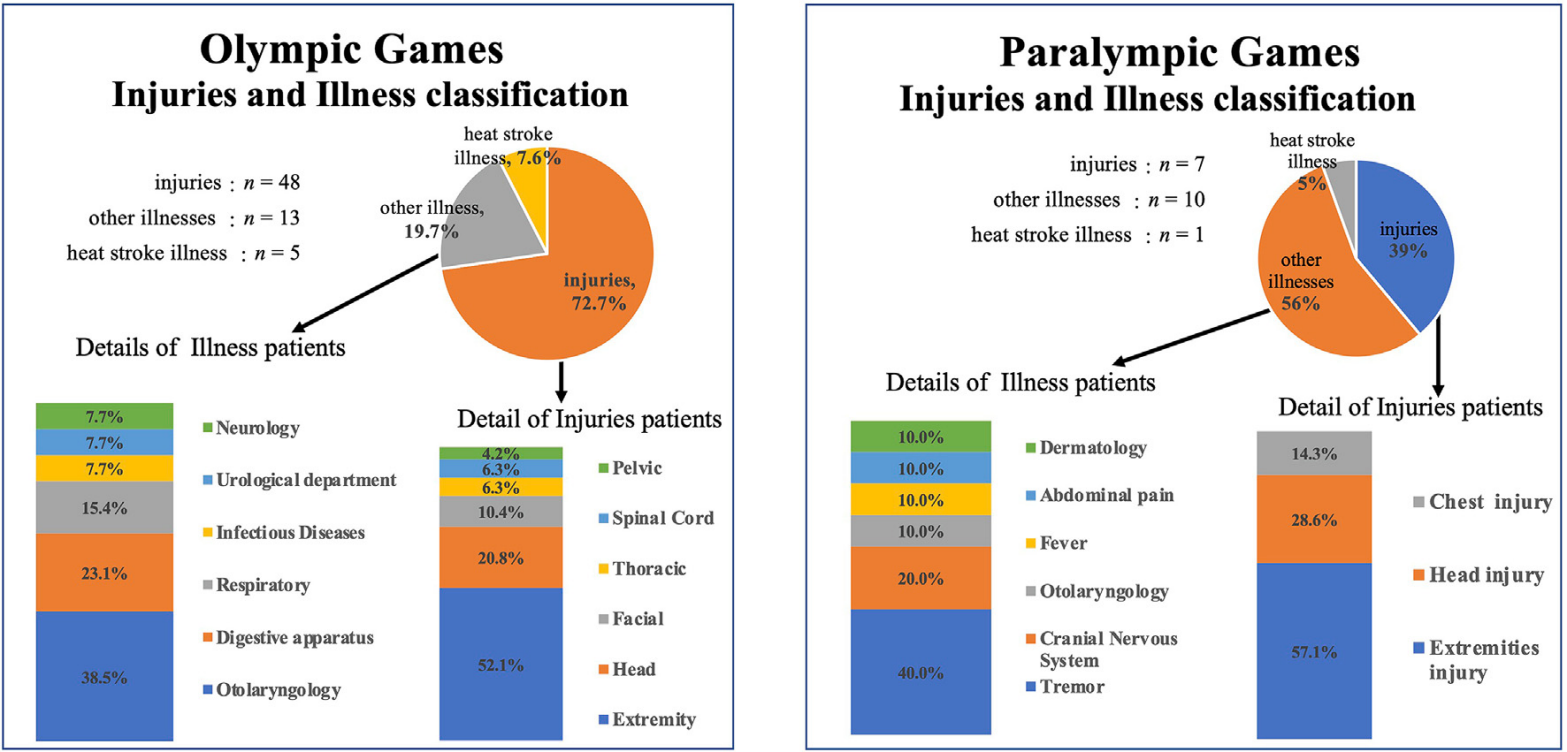
Detailed rainfall of injuries and other illnesses occurring at the Olympic and Paralympic Games.

Details of the classification of injuries and other illnesses during external medical visits at the Olympic and Paralympic Games are shown in Fig. 2.

In the Olympic Games, the most common injuries (*n* ​= ​48) were injuries to the extremities (*n* ​= ​25, 52.1%), followed by injuries to the head (*n* ​= ​11, 22.9%), face (*n* ​= ​6, 12.5%), cervical spine (*n* ​= ​3, 6.3%), and chest (*n* ​= ​3, 6.3%). Regarding other illnesses (*n* ​= ​13), otolaryngology (*n* ​= ​5, 38.5%) was the most common illness, followed by gastrointestinal diseases in three cases (23.1%) and respiratory diseases in two cases (15.4%).

On the other hand, the most common Paralympic Games injuries (*n* ​= ​7) were injuries to the extremities (*n* ​= ​4, 57.1%), with two (28.6%) to the head and one (14.3%) to the chest. Among other illnesses (*n* ​= ​10), convulsions (*n* ​= ​4, 44.4%) were the most common, followed by cranial nerve disease in two cases (20.0%).

## 4 Discussion

The purpose of this study was to analyze the occurrence of injuries and other illnesses that required visits to outside facilities during the Tokyo 2020 Games. The results showed that 66 cases (0.6% of total athlete participation) occurred during the Olympic Games, and 18 (0.4%) cases occurred during the Paralympic Games. In the Olympic Games, more than 70% of cases occurred due to trauma. When classified according to the types of sports, the highest number of cases occurred in soccer. On the other hand, In the Paralympics Games, 50% is an illness, occurring in swimming, cycling, and track and field events.

### 4.1 Incidence, trends, and severity of injuries

Previous reports of injuries and other illnesses during the Summer Olympic Games indicate that in the 2016 Rio de Janeiro Games, injuries occurred in 8% of all athletes and other illnesses occurred in 5% of athletes.^2^ In the 2012 London Games, injuries occurred in 11% of all athletes, while other illnesses occurred in 7% of athletes.^5^ However, there are no detailed reports on visits to outside facilities, excluding the medical unit at the games; hence, this aspect is not clear. In the Olympic Game period studied in this study, 72.7%, or more than half, of the visits to outside facilities were due to injuries. Of the five cases of injuries, two were cervical cord injuries in basketball competitions, and the other injuries included head injuries in BMX, limb injuries in judo, and limb and head injuries in bicycle track events. We observed a tendency for injuries to be more severe in sports where there is contact with opponents or when falls from vehicles are involved, such as cycling or BMX games.^10^ On the other hand, seven cases (41.1%) of injuries occurred at the Paralympic Games, which involved four cases in cycling road races, two cases in powerlifting, and one case in track and field events; of these cases, four required hospitalizations within 7 days. Three of the four cases that required hospitalization occurred during cycling; moreover, similar to the Olympics, cycling tended to be the most common sport involved. It is important for nearby outside facilities to have a system that enables prompt examination, such as CT and MRI, and specialists in the field of orthopedics, in anticipation of severe trauma during sports.

### 4.2 Incidence, trends, and severity of other illnesses

Regarding other illnesses, outbreaks of infectious diseases have been a concern at large international events, such as the Olympics. In the Rio Games, it was reported that 56% of other illnesses outbreaks among athletes were caused by infectious diseases.^2^^,^^11^ However, our study found that, excluding Coronavirus disease (COVID-19), only one case of an infectious disease (a malarial infection) resulted in a visit to an outside medical facility. The most common illness was otorhinolaryngological disease, which is thought to have occurred because the clinics at each venue mainly provided acute care and physiotherapy services, and were unable to immediately treat otorhinolaryngological diseases, necessitating visits to outside facilities. In the future, outside facilities supporting the convention should establish hospitals that can handle general emergencies and the otorhinolaryngology area.

In the Paralympic Games, the incidence of other illnesses tended to be higher than that of injuries,^12^^,^^13^ and the incidence of sudden other illnesses was higher than the incidence of injuries. It is suggested that in the Paralympic Games, the clinical history of the athletes revealed that injuries and other illnesses were more likely to occur due to the influence of underlying impairments, including original spinal cord injuries and neurological abnormalities.^11^ According to reports from the London Games, the most common illness during the Paralympic Games occurred in the respiratory system, followed by cases involving the skin and digestive system.^13^^,^^14^ However, regarding other illnesses (*n* ​= ​10) at the current Games, convulsions (*n* ​= ​4, 44.0%) occurred most frequently, probably due to temporary abnormalities in the brain regions. It is believed that tremors occurred because of temporary abnormalities in the brain parenchyma. In the Paralympics, more cases of illness than of injuries use outside medical facilities. With this in mind, it is important to share the athlete's medical history with outside facilities for treatment.

Collection and analysis of the reasons of injuries and illnesses will provide guidance for the medical support of future Olympic and Paralympic Games.

### 4.3 Occurrence of HSI

Although it has been reported that the number of HSI outbreaks was high, especially in marathon events^,^^15^, ^16^, ^17^ only five cases of heat stroke resulted in visits to outside facilities in this study, including three cases in marathons, one in track and field, and one in judo. However, all cases required only medical examinations, and none of the athletes was hospitalized. In accordance with the recommendations of the IOC Adverse Weather Impact Expert Working Group for the Olympic Games Tokyo 2020,^9^^,^^18^ thorough response measures were taken in advance, including an initial screening system at each venue and the administration of an oral rehydration solution. Marathon and walking race competitions were also influenced by the fact that they were held in Sapporo, a city in the northern region of Japan, considering the heat of the Tokyo metropolitan area. This is believed to have prevented serious cases of HSI that required hospitalization.

### 4.4 Limitations

The limitations of this study are as follows: (1) it is a descriptive study that presented the details of visits to outside facilities during the Tokyo 2020 Games and does not infer causal relationships; (2) identification of injuries and other illnesses, as well as data collection were conducted by physicians in Japan in accordance with IOC standards; hence, their identifications were not uniform; (3) since the games were held in Japan, standards such as emergency transport systems and field triage standards, such as primary, secondary, and tertiary emergency care, differed from those of other countries; and (4) since the Games were held during the COVID-19 pandemic, medical support differed significantly from those of other games.

## 5 Conclusion

This study determined the incidence of injuries and other illnesses that occurred during the Tokyo 2022 Games among athletes who visited other facilities. In total, 66 (5.8/1 000) and 18 (4.1/1 000) athletes visited external medical facilities during the Olympic and Paralympic Games, respectively. During the Olympic Games, injuries accounted for 70% of all cases, but only three (0.05%) cases were severe and required hospitalization for more than 1 week. During the Paralympic Games, other illnesses accounted for nearly half of all cases. This study provides details on the extent of injuries and other illnesses that were transferred to outside facilities, which has not been evident in previous games.

## Submission statement

All authors have read and agree with manuscript content. This manuscript has not been published and is not under consideration for publication elsewhere.

## Ethical approval statement

In this study, medical data were obtained from the Organizing Committee and used after de-identification of the subjects’ personal information. Additionally, an application for secondary use of the data was submitted to the Ethics Committee on Research Involving Human Subjects of Kokushikan University, and approval for the implementation of the research was obtained (approval number: 21 ​033).

## Authors' Contribution

**Shuji Sakanashi:** Writing – original draft. **Hideharu Tanaka:** Writing – original draft, Writing – review & editing. **Hiroyuki Yokota:** Supervision. **Yasuhiro Otomo:** Supervision. **Tomohiko Masuno:** Investigation. **Kousuke Nakano:** Investigation. **Junichi Inoue:** Investigation. **Manabu Sugita:** Investigation. **Takahiko Tokunaga:** Investigation. **Nagisa Kato:** Investigation. **Tomoya Kinoshi:** Investigation. **Hironori Inoue:** Investigation. **Hiroto Numata:** Investigation. **Koshi Nakagawa:** Formal analysis. **Ryo Sagisaka:** Formal analysis. **Shota Tanaka:** Writing – review & editing. **Tetsuya Miyamoto:** Supervision. **Takao Akama:** Supervision.

## Confict of interest

The authors declare that they have no known competing financial interests or personal relationships that could have appeared to influence the work reported in this paper.
